# Household-specific physical activity levels and energy intakes according to the presence of metabolic syndrome in Korean young adults: Korean National Health and nutrition examination survey 2016–2018

**DOI:** 10.1186/s12889-022-12852-3

**Published:** 2022-03-10

**Authors:** Young-Jun Lee, Yeon-Hee Park, Jung-Woo Lee, Eun-Sook Sung, Hyun-Seob Lee, Jonghoon Park

**Affiliations:** 1 Exercise Nutrition and Biochemistry Laboratory, Department of Physical Education, Korea University, 145 Anam-ro, Seongbuk-gu, Seoul, 02841 Republic of Korea; 2 Department of Physical Education, Graduate School of Education, Korea University, 145 Anam-ro, Seongbuk-gu, Seoul, 02841 Republic of Korea; 3 Department of Home Economics Education, Korea University, 145 Anam-ro, Seongbuk-gu, Seoul, 02841 Republic of Korea

**Keywords:** Energy intake, Metabolic syndrome, Physical activity, Single-person household

## Abstract

**Background:**

Participation in exercise, and dietary and nutritional intakes have an impact on the risk and prevalence of metabolic syndrome (MetS), but these effects may differ according to whether a person lives alone or in a multi-person household. We analyzed differences in physical activity (PA) levels and energy intake according to household-type and MetS presence among young adults, to investigate the relationships among these factors.

**Methods:**

Data of 3974 young adults (aged > 19 years and < 40 years) were obtained from the Korean National Health and Nutrition Examination Survey (2016–2018). We analyzed PA levels (occupational and recreational PA, and transport) and energy intake (total, carbohydrate, protein, and fat).

**Results:**

Logistic regression data showed that low PA levels and higher energy intake were associated with MetS incidence and its components in young adults, after adjusting for body mass index, smoking, household-type, and sex. Overall, there was no significant difference in PA level between the MetS and non-MetS group. The total energy intake was higher in the MetS than in the non-MetS group (*p* <  0.05). These results were similar to those found in multi-person households. In single-person households, the MetS group had significantly lower PA levels (*p* <  0.01) and total energy intake (*p* <  0.05) than the non-MetS group.

**Conclusions:**

We found significant association among low PA levels, high energy intake, and MetS components in young Korean adults, but with patterns differing according to household type. Energy intake was higher in young adults with than those without MetS, who lived in multi-person households, while young adults with MetS who lived alone had lower PA levels and lower energy intake than those without MetS. These findings highlight the need for different approaches of implementing PA and nutrition strategies according to the type of household in order to prevent MetS.

## Introduction

The single-person household is the fastest growing type of household in many regions of the world, due to changes in institutional arrangements, demographic behaviors, and labor migration in the past few decades [1]. Not only widowed people, but also many young adults who have never been married, now live alone. The family structure in Korea has changed from the traditional large extended family to the nuclear family, due to industrialization and urbanization, and recently, the number of single-person households living alone in Korea has increased rapidly [2]. According to the National Statistical Office, the percentage of single-person households has increased from 15.5% in 2000, to 29.3% in 2018, and is estimated to reach 34.3% by 2035 [3]. Recently, the proportion of young adults living in single-person households has increased remarkably worldwide [4].

According to the Korean National Health and Nutrition Examination Survey (KNHANES), the incidence of metabolic syndrome (MetS) has increased by 0.6% every 10 years since 1998 [5]. Additionally, recent studies in Korea have found that single-person households are more susceptible than multi-person households to insufficient physical activity (PA) and unhealthy eating practices [6]. The amount of PA has decreased, with a decrease in moderate PA and walking, without an increase in high-intensity PA [7, 8]. Low levels of PA, in turn, contribute to the increase in MetS incidence.

In conjunction with social changes in Korea, the dietary behavior of Koreans has also changed rapidly. With increasing numbers of single-person households, eating alone has become a social concern. Some previous studies reported that eating alone was associated with various health problems. Eating alone was related to a reduced calorie intake and a less-varied diet [9, 10], and it could be a direct risk factor for MetS [11].

The level of PA, the degree of participation in exercise, and dietary and nutritional intakes have an impact on the risk for and prevalence of MetS. In a recent comparative analysis, among 66,211 older individuals (aged 60 years or more) in Korea, single-person households are considered to have worse overall health behaviors, such as exercise behaviors and nutritional behaviors, than multi-person households [12]. According to KNHANES data (2013–2015) of 2903 subjects ≥ aged 65 years, single-person households had worse nutrient intake overall, and had an increased prevalence of MetS [13].

Research on housing and MetS of young people, particularly young single-person households, has been scant internationally. Moreover, there have been few studies about household type-specific PA and energy intake in young adults according to the presence of MetS. Thus, it is essential to examine the relationship between these factors in a large population. However, even adequately-sized cohort studies evaluating the PA levels and energy intakes according to the household types and presence of MetS are not appropriate, because these factors may vary by young adults’ lifestyles and household types. Therefore, this study aimed to analyze the differences in PA levels and energy intake by household type and the presence of MetS in a young adult Korean population, based on data from the 7th Korea National Health and Nutrition Examination Survey (2016–2018).

## Materials and methods

### Sample and design

This study used cross-sectional data from the KNHANES from 2016 to 2018, which was conducted by the Korea Centers for Disease Control and Prevention (KCDC). This data is updated every 3 years. Therefore, we used the most up-to-date data available. The details of the study design and data resource profiles followed the methods described in the Guidelines for Use of the KNHANES Raw Data and the Final Report of the sampling frame [14]. The KNHANES consists of a health interview survey, a nutrition survey, and a health examination, and is conducted according to the Declaration of Helsinki. This survey was approved by the Institutional Review Board of the Korea Centers for Disease Control and Prevention (reference number: 2018-01-03-P-A). All participants in the survey signed an informed consent form.

**Fig. 1:**
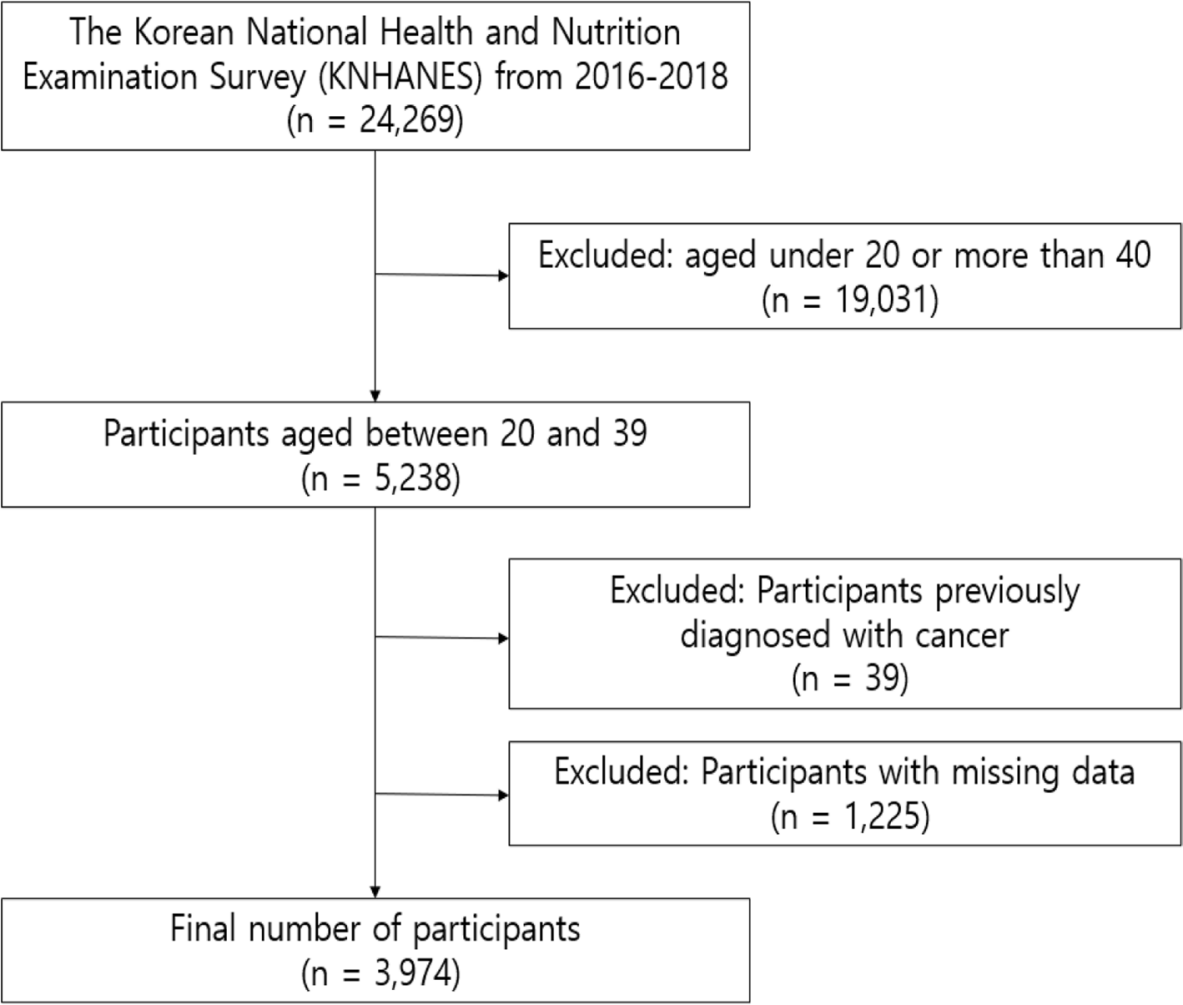
Flow diagram for the selection of study participants

Between 2016 and 2018, 24,269 individuals completed the health interview survey, nutrition survey, and health examination. Among them, 19,031 people aged under 20 years or more than 40 years were excluded, leaving 5238 people between the ages of 20 years and 39 years. Participants previously diagnosed with cancer (gastric, liver, colon, breast, cervical, lung, thyroid, and other cancers), and those with missing data (anthropometric, health examination, and PA data) were excluded (Fig. 1). In total, 3974 young people were finally included in this study.

### Measures

The presence or absence of MetS was determined using measurements of waist circumference, blood pressure, fasting blood glucose levels, triglyceride levels, and HDL-C levels. These MetS measures were determined by survey. The KHANES data presented the PA variables of occupational activity, recreation activity, and transport in metabolic equivalents (MET)-minutes/week based on the Global Physical Activity Questionnaire (GPAQ). In addition, the KHANES data presented the nutrition outcomes of the amount of intake by the 24-h recall method for each individual by interviewing the target households in person. The analyzed characteristics of the participants according to household are shown in Table 1.

**Table 1:** Descriptive characteristics of the participants

	Total (***n*** = 3974)			Multi-person Households (***n*** = 3619)			Single-person Households (***n*** = 355)		
	Non-MetS (***n*** = 3477)	MetS (***n*** = 497)	*p*-Value	Non-MetS (***n*** = 3169)	MetS (***n*** = 450)	*p*-Value	Non-MetS (***n*** = 308)	MetS (***n*** = 47)	*p*-Value
Age (years)	29.6 ± 0.2	32.5 ± 0.3	< 0.001***	29.7 ± 0.2	32.7 ± 0.3	< 0.001***	28.7 ± 0.3	31.3 ± 0.9	0.008**
Height (cm)	167.9 ± 0.2	172.3 ± 0.4	< 0.001***	167.7 ± 0.2	172.1 ± 0.4	< 0.001***	170.0 ± 0.4	173.9 ± 1.0	< 0.001***
Body weight (kg)	64.9 ± 0.3	86.4 ± 0.8	< 0.001***	64.6 ± 0.3	85.7 ± 0.9	< 0.001***	66.8 ± 0.8	91.9 ± 1.7	< 0.001***
BMI (kg/m^2^)	22.9 ± 0.1	29.0 ± 0.2	< 0.001***	22.9 ± 0.1	28.8 ± 0.3	< 0.001***	23.0 ± 0.3	30.3 ± 0.5	< 0.001***
Alcohol (%)	67.7	72.3	0.105	66.5	71.1	0.131	77.7	82.2	0.469
Smoking (%)	21.8	39.5	< 0.001***	20.5	39.6	< 0.001***	32.9	38.9	0.516

Values are expressed as means standard errors; BMI, body mass index; Alcohol, Percentage of drinking at least once a month in the past year; Smoking, Percentage of smoking five packs (100 cigarettes) or more in their lifetime and current smoking statuse; MetS, metabolic syndrome

^*^p <  0.05

^**^p <  0.01

^***^p <  0.001

### Metabolic syndrome

The diagnosis of MetS was based on the new harmonized guidelines of the National Cholesterol Education Program-Adult Treatment Panel III and the American Heart Association and the National Heart Lung and Blood Institutes [15, 16]. For waist circumference, we followed the criteria suggested by the Korean Society for the Study of Obesity [17]. If three or more of the following five criteria were met, participants were classified as having MetS: waist circumference > 90 cm (men) or > 85 cm (women); systolic blood pressure > 130 mmHg or diastolic blood pressure > 85 mmHg; fasting triglyceride (TG) levels > 150 mg/dL; fasting HDL-C levels < 40 mg/dL (men) or < 50 mg/dL (women); and fasting glucose levels > 100 mg/dL.

### Physical activity

The GPAQ comprises 16 questions grouped to capture PA undertaken in different behavioral domains: work, transport, and recreational activities. It analyzes five domains of PA: vigorous-intensity work, moderate-intensity work, transport, vigorous-intensity recreation, and moderate-intensity recreation. The participants responded freely to the five domains without any additional options regarding how many times a week and how many minutes per day they did the activity. The World Health Organization (WHO) GPAQ analysis guidelines were used to analyze GPAQ data [18]. We estimated that, compared to while sitting quietly, a person’s caloric consumption was four times higher when they were being moderately active and eight times higher when they were being vigorously active. Therefore, when calculating the total energy expenditure of an individual using GPAQ data, four METs were allocated to the time spent in moderate activity and eight METs to the time spent in vigorous activity, and the details are as follows:

- Vigorous intensity activity: occupational (MET) = 8.0 × vigorous intensity physical activity (day/week) × 1-day vigorous intensity physical activity (minutes/day)
- Moderate intensity activity: occupational (MET) = 4.0 × moderate intensity physical activity (day/week) × 1-day moderate intensity physical activity (minutes/day)
- Vigorous intensity activity: recreational (MET) = 8.0 × vigorous intensity physical activity (day/week) × 1-day vigorous intensity physical activity (minutes/day)
- Moderate intensity activity: recreational (MET) = 4.0 × moderate intensity physical activity (day/week) × 1-day moderate intensity physical activity (minutes/day)
- Transport (MET) = 4.0 × transport physical activity (day/week) × 1-day transport physical activity
- Total Physical Activity (MET) = vigorous intensity activity: occupational + moderate intensity activity: occupational + vigorous intensity activity: recreational + moderate intensity activity: recreational + transport.

PA levels were classified into four groups: inactive (0–249 MET min/week), somewhat active (250–499 MET min/week), active (500–999 MET min/week), and very active (> 1000 MET min/week). These cut-off points are based on their equivalence to the following PA thresholds: 250 MET min/week represents an energy expenditure dose equivalent to half of threshold; 500 MET min/week is equivalent to the minimal threshold; and 1000 MET min/week is equivalent to twice the minimal threshold [19].

### Energy intake

The nutrition outcomes were obtained by the 24-h recall method for each individual by interviewing the target households in person. The nutrition survey data were collected by trained dietitians in the homes of the participants 1 week after the health interview and health examination. The daily energy intake was calculated using the Korean Foods and Nutrients Database of the Rural Development Administration. The following items were included in the analyses: total energy intake, carbohydrate intake, protein intake, and fat intake per day. The energy intake data was converted to kcal. Carbohydrates and proteins were calculated as 4 kcal per 1 g, and fat was calculated as 9 kcal per 1 g. We conducted a sensitivity analysis to identify low-energy reporters/over-energy reporters using the Goldburg cut-off. In the case of 1 recall day, the lower 95% cut-off was 0.87, and the upper 95% cut-off was 2.75 [20]. Energy intake was categorized by dividing the ratio with estimated energy requirement (EER). EER is the average dietary energy intake that is predicted to maintain energy balance in healthy, normal weight individuals of the defined age, gender, weight, height, and level of physical activity consistent with good health. We used EER based on the Institute of Medicine (IOM) equations, by body mass index, age, and sex. Then, we selected a Physical Activity Levels (PAL) to estimate energy requirements and predict the ranges of physical activity levels. Values less than 20% EER (< 0.8) were considered as lower intake, whereas values above 1.2 as higher intake [ 21].

### Statistical analysis

Continuous variables were presented as means and standard errors. Normality of the distribution of all outcome-variable data was verified using the Kolmogorov–Smirnov test. An independent *t*-test was used to analyze risk factors for MetS, as well as PA levels and energy intakes between the non-MetS and MetS group. Two-way analyses of variance (ANOVA) were used to analyze the differences in risk factors for MetS, PA levels, energy intakes between participants with and without MetS, and between single-person and multi-person households. Partial eta-squared (η^2^) values were calculated to represent effect sizes. If a significant interaction effect was found by two-way ANOVA, a Bonferroni post-hoc test was used to compare the household-specificity of dependent variables in each group (with and without MetS) separately. Moreover, the relationships between PA levels or energy intake and MetS were determined using logistic regression analysis after controlling for covariates. Logistic regression analysis findings were presented as odds ratios (ORs) and their associated 95% confidence intervals (CIs). Statistical analyses were performed using SPSS version 25.0 for Windows (IBM Corp., Armonk, NY, USA). The level of significance was set at 0.05.

## Results

The average values of PA levels and energy intake factors are shown in Table 2 and the ORs for MetS and MetS components according to PA levels and energy intake are presented in Table 3. The ORs were adjusted for body mass index, smoking, household-type, and sex in model 2. We found that an “Active” PA level was associated with low HDL-C levels (OR = 0.76, 95%CI = 0.61–0.93). A PA level of “Very active” was associated with a lower MetS incidence (OR = 0.55, 95%CI = 0.39–0.77), larger waist circumference (OR = 0.64, 95%CI = 0.43–0.93), high triglyceride (TG) levels (OR = 0.69, 95%CI = 0.56–0.87), and low levels of high-density lipoprotein C (HDL-C) (OR = 0.72, 95%CI = 0.59–0.88). We also found that “Moderate energy intake” levels were associated with a larger waist circumference (OR = 1.46, 95%CI = 1.05–2.04) and high glucose levels (OR = 1.23, 95%CI = 1.02–1.63). “Higher energy intake” levels were associated with the high TG levels (OR = 1.33, 95%CI = 1.03–1.70), low HDL-C levels (OR = 0.77, 95%CI = 0.61–0.97), and high glucose levels (OR = 1.39, 95%CI = 1.01–1.92).

**Table 2:** Classification of physical activity levels and energy intake

Factors	Total	***n***	Multi-person Households	***n***	Single-person Households	***n***
Physical activity factors	MET min/Week (Mean ± SE)					
Inactive (0–249 MET min/week)	39.6 ± 2.5	1354	40.0 ± 2.6	1255	35.4 ± 7.6	99
Somewhat active (250–499 MET min/week)	406.9 ± 3.2	464	406.1 ± 3.3	428	415.9 ± 11.3	36
Active (500–999 MET min/week)	750.7 ± 5.3	834	752.5 ± 5.8	746	739.2 ± 12.5	88
Very active (> 1000 MET min/week)	2775.1 ± 100.2	1322	2754.4 ± 97.8	1190	2944.4 ± 345.2	132
Energy intake factors	Energy intake/EER (Mean ± SE)					
Lower energy intake (energy intake/EER < 0.8)	0.6 ± 0.0	2067	0.6 ± 0.0	1884	0.5 ± 0.0	183
Moderate energy intake (0.8 ≤ energy intake/EER ≤ 1.2)	1.0 ± 0.0	1288	1.0 ± 0.0	1163	1.0 ± 0.0	125
Higher energy intake (energy intake/EER > 1.2)	1.5 ± 0.0	619	1.5 ± 0.0	572	1.4 ± 0.0	47

Values are expressed as means ± standard errors

*MET* Metabolic equivalents of task, *EER* Estimated energy requirements

**Table 3:** Odds ratio (95% CI) for MetS and MetS components according to physical activity levels and energy intake

Factors	MetS	Large Waist Circumference	High Triglycerides	Low HDL-C	High Blood Pressure	High Glucose
**Model 1**^a^						
Physical activity factors						
Inactive (*n* = 1354)	1.00 (reference)	1.00 (reference)	1.00 (reference)	1.00 (reference)	1.00 (reference)	1.00 (reference)
Somewhat active (*n* = 464)	0.79 (0.54–1.16)	0.89 (0.64–1.23)	0.69 (0.51–0.94)*	0.80 (0.59–1.09)	0.71 (0.48–1.05)	0.84 (0.57–1.24)
Active (*n* = 834)	0.86 (0.64–1.16)	0.927 (0.711–1.207)	0.78 (0.62–0.97)*	0.77 (0.63–0.94)*	1.10 (0.83–1.46)	0.99 (0.72–1.35)
Very active (*n* = 1322)	0.84 (0.63–1.12)	1.02 (0.81–1.29)	0.88 (0.72–1.08)	0.74 (0.61–0.89)**	1.15 (0.89–1.49)	0.96 (0.75–1.22)
Energy intake factors						
Lower energy intake (energy intake/EER < 0.8)	1.00 (reference)	1.00 (reference)	1.00 (reference)	1.00 (reference)	1.00 (reference)	1.00 (reference)
Moderate energy intake (0.8 ≤ energy intake/EER ≤ 1.2)	0.85 (0.66–1.10)	0.77 (0.63–0.93)**	0.92 (0.74–1.14)	0.90 (0.76–1.07)	0.90 (0.73–1.12)	1.07 (0.84–1.36)
Higher energy intake (energy intake/EER > 1.2)	0.84 (0.62–1.13)	0.77 (0.58–1.02)	1.16 (0.93–1.46)	0.68 (0.54–0.85)**	0.86 (0.64–1.15)	1.16 (0.86–1.55)
**Model 2**^b^						
Physical activity factors						
Inactive (*n* = 1354)	1.00 (reference)	1.00 (reference)	1.00 (reference)	1.00 (reference)	1.00 (reference)	1.00 (reference)
Somewhat active (*n* = 464)	0.91 (0.58–1.41)	1.10 (0.68–1.80)	0.75 (0.53–1.06)	0.80 (0.58–1.11)	0.77 (0.51–1.18)	0.92 (0.62–1.36)
Active (*n* = 834)	0.86 (0.59–1.24)	1.05 (0.65–1.70)	0.78 (0.60–1.01)	0.76 (0.61–0.93)**	1.12 (0.82–1.52)	1.01 (0.74–1.39)
Very active (*n* = 1322)	0.55 (0.39–0.77)***	0.64 (0.43–0.93)*	0.69 (0.56–0.87)**	0.72 (0.59–0.88)**	0.90 (0.67–1.19)	0.81 (0.63–1.05)
Energy intake factors						
Lower energy intake (energy intake/EER < 0.8)	1.00 (reference)	1.00 (reference)	1.00 (reference)	1.00 (reference)	1.00 (reference)	1.00 (reference)
Moderate energy intake (0.8 ≤ energy intake/EER ≤ 1.2)	1.20 (0.88–1.64)	1.46 (1.05–2.04)*	1.05 (0.83–1.32)	1.02 (0.85–1.22)	1.07 (0.86–1.35)	1.23 (1.02–1.63)*
Higher energy intake (energy intake/EER > 1.2)	1.19 (0.81–1.75)	1.58 (0.94–2.65)	1.33 (1.03–1.70)*	0.77 (0.61–0.97)*	1.01 (0.71–1.42)	1.39 (1.01–1.92)*

Data presented as odds ratio (95% confidence intervals (CIs))

*OR* odds ratio, *MetS* metabolic syndrome, *HDL-C* high-density lipoprotein cholesterol, *EER* estimated energy requirements

^a^Model 1: crude

^b^Model 2: adjusted for sex, smoking, household type, and body mass index

^*^p <  0.05 vs. reference

^**^p <  0.01 vs. reference

^***^p <  0.001 vs. reference

The differences in variables considered risk factors for MetS, according to the presence or absence of MetS and according to households are presented in Table 4. In the total group, young individuals with MetS had significantly higher values of risk factors for MetS than did those in the non-MetS group (all variables, *p* < 0.001). The interaction between the presence or absence of MetS and household type (single- or multi-person) was statistically significant for waist circumference, TG, HDL-C, diastolic blood pressure (DBP), fasting blood glucose (all *p* < 0.001). The Bonferroni post-hoc test showed the following group differences: Individuals with MetS living in multi-person households had significantly larger waist circumference, TG, DBP, fasting blood glucose (all *p* < 0.001), but lower HDL-C (*p* < 0.001), than their counterparts without MetS. Individuals with MetS who lived alone had significantly larger waist circumference, TG, HDL-C, DBP, and fasting blood glucose than those without MetS (all *p* < 0.001). Individuals with MetS living as single persons had statistically significantly greater waist circumference and higher DBP (both *p* < 0.01) than individuals with MetS living in multi-person households.

**Table 4:** Metabolic Syndrome Components

Factors	Group	Total	Household type		ANOVA			
			Multiple	Single	***F***-value		*p* -Value (η^2^)	Power
Waist circumference (cm)	Non-MetS MetS *p*-value	77.9 ± 0.2 95.63 ± 0.5 < 0.001***	77.8 ± 0.2 95.2 ± 0.6†	78.6 ± 0.7 99.3 ± 1.1†‡	H M H × M	62.542*** 1489.340*** 87.608***	0.000(0.006) 0.000(0.068) 0.000(0.008)	1.000 1.000 1.000
TG (mg/dL)	Non-MetS MetS *p*-value	102.8 ± 1.7 242.7 ± 9.5 < 0.001***	102.2 ± 1.9 243.5 ± 9.9†	107.8 ± 2.9 236.1 ± 24.6†	H M H × M	2.390 748.808*** 9.114***	0.092(0.000) 0.000(0.037) 0.000(0.001)	0.485 1.000 0.976
HDL-C (mg/dL)	Non-MetS MetS *p*-value	54.9 ± 0.3 40.9 ± 0.4 < 0.001***	55.0 ± 0.2 40.7 ± 0.4†	53.8 ± 0.9 42.2 ± 1.4†	H M H × M	0.022 552.453*** 22.619***	0.978(0.000) 0.000(0.027) 0.000(0.002)	0.053 1.000 1.000
SBP (mmHg)	Non-MetS MetS *p*-value	109.5 ± 0.3 123.0 ± 0.6 < 0.001***	109.1 ± 0.2 122.6 ± 0.7	112.4 ± 0.9 125.9 ± 2.3	H M H × M	176.024*** 451.997*** 0.485	0.000(0.017) 0.000(0.022) 0.485(0.616)	1.000 1.000 0.130
DBP (mmHg)	Non-MetS MetS *p*-value	72.6 ± 0.2 85.1 ± 0.5 < 0.001***	72.4 ± 0.2 84.6 ± 0.5†	74.8 ± 0.6 88.6 ± 1.4†‡	H M H × M	43.256*** 629.397*** 61.583***	0.000(0.004) 0.000(0.030) 0.000(0.006)	1.000 1.000 1.000
Fasting glucose (mg/dL)	Non-MetS MetS *p*-value	90.3 ± 0.3 108.0 ± 1.5 < 0.001***	90.3 ± 0.3 108.5 ± 1.6†	90.4 ± 0.5 104.2 ± 2.7†	H M H × M	130.082*** 359.141*** 7.636***	0.000(0.013) 0.000(0.018) 0.000(0.001)	1.000 1.000 0.948

Values are expressed as means standard errors

Main effect = H (Household) and M (Metabolic syndrome), Interaction effect = H × M (Household × Metabolic syndrome)

Two-way analysis of variance following Bonferroni post-hoc test

*MetS* metabolic syndrome, *TG* triglyceride, *HDL-C* high-density lipoprotein cholesterol, *SBP* systolic blood pressure, *DBP* diastolic blood pressure, *HbA1c* glycated hemoglobin

^*^p < 0.05

^**^p < 0.01

^***^p < 0.001

^†^*p* < 0.001 compared with Non-MetS values

^‡^*p* < 0.01 compared with Multiple values

The differences in PA levels according to the presence or absence of MetS and according to household type are presented in Table 5. In the total group, there was no significant difference in PA levels between young individuals with and without MetS. For the PA aspects of “Occupational vigorous” (*p* < 0.05), “Transport” (*p* < 0.05), and “Total PA” (*p* < 0.01), there was a significant interaction between the presence or absence of MetS and household type. The Bonferroni post-hoc test showed the following group differences: Individuals with MetS living in multi-person households showed significantly higher “Occupational vigorous” PA (*p* < 0.05) than their counterparts without MetS. Those with MetS who lived alone demonstrated significantly lower “Transport” and “Total” PA (all *p* < 0.01) than their counterparts without MetS. There was no significant difference between those with MetS living in single-person households and those with MetS living in multi-person households.

**Table 5:** Levels of physical activity

Physical Activity (MET min/week)	Group	Total	Household type		ANOVA			
			Multiple	Single	***F***-value		*p* -Value (η^2^)	Power
Occupational vigorous	Non-MetS MetS *p*-value	76.7 ± 15.5 132.4 ± 47.8 0.271	65.1 ± 12.8 148.5 ± 53.5†	176.0 ± 86.9 1.7 ± 1.7	H M H × M	3.765* 0.836 4.575*	0.023(0.000) 0.361(0.000) 0.010(0.000)	0.689 0.150 0.778
Occupational moderate	Non-MetS MetS *p*-value	214.6 ± 23.9 234.7 ± 45.5 0.688	214.0 ± 25.0 251.8 ± 51.0	219.3 ± 61.2 95.6 ± 45.8	H M H × M	29.891*** 0.850 1.621	0.000(0.003) 0.357(0.000) 0.198(0.000)	1.000 0.152 0.345
Transport	Non-MetS MetS *p*-value	524.2 ± 17.6 474.2 ± 43.9 0.253	514.5 ± 17.6 490.2 ± 48.1	607.7 ± 69.5 344.0 ± 70.6††	H M H × M	26.954*** 9.888** 3.559*	0.000(0.003) 0.002(0.000) 0.028(0.000)	1.000 0.882 0.663
Recreational vigorous	Non-MetS MetS *p*-value	212.9 ± 17.0 191.9 ± 39.6 0.628	209.3 ± 17.4 194.2 ± 44.6	243.5 ± 42.6 172.8 ± 68.3	H M H × M	30.567*** **3.324** 0.991	0.000(0.003) 0.068(0.000) 0.371(0.000)	1.000 0.446 0.224
Recreational moderate	Non-MetS MetS *p*-value	181.8 ± 9.0 149.7 ± 23.2 0.206	183.0 ± 9.8 147.1 ± 23.8	171.1 ± 22.6 171.4 ± 55.2	H M H × M	10.371*** 1.648 0.243	0.000(0.001) 0.199(0.000) 0.784(0.000)	0.988 0.250 0.088
Total physical Activity	Non-MetS MetS *p*-value	1210.2 ± 48.5 1182.9 ± 105.8 0.812	1186.0 ± 46.6 1231.8 ± 116.1	1417.7 ± 168.0 785.4 ± 172.7††	H M H × M	64.415*** 9.312** 5.062**	0.000(0.006) 0.002(0.000) 0.006(0.000)	1.000 0.862 0.820

Values are expressed as means standard errors

Main effect = H (Household) and M (Metabolic syndrome), Interaction effect = H × M (Household × Metabolic syndrome)

Two-way analysis of variance following Bonferroni post-hoc test

*MetS* metabolic syndrome

^*^p < 0.05

^**^p < 0.01

^***^p < 0.001

^†^*p* < 0.05

^††^*p* < 0.01 compared with Non-MetS values

Table 6 shows the differences in energy intake according to the presence or absence of MetS and according to household type. In the total group, young individuals with MetS had significantly higher energy intake than those without MetS (total energy intake and protein intake, *p* < 0.001; carbohydrate intake, *p* < 0.05). For “Total energy intake”, “Protein intake”, “Fat intake” (all *p* < 0.001), and for “Carbohydrate intake” (*p* = 0.001), there was a significant interaction between the presence or absence of MetS and household type. The Bonferroni post-hoc test showed the following group differences: Individuals with MetS living in multi-person households had significantly higher total energy intake, protein intake, fat intake (all *p* < 0.001), and carbohydrate intake (*p* < 0.01) than those without MetS who lived in multi-person households. Persons with MetS who lived as single persons had significantly lower total energy intake (*p* < 0.05) and fat intake (*p* < 0.01) than those without MetS who lived alone. Those with MetS who lived alone had a significantly lower total energy intake and fat intake (*p* < 0.01) than those with MetS who lived in a multi-person household.

**Table 6:** Energy intake

Factors	Group	Total	Household type		ANOVA			
			Multiple	Single	***F***-value		*p* -Value (η^2^)	Power
Total energy intake (kcal)	Non-MetS MetS *p*-value	2149.0 ± 19.4 2396.7 ± 58.9 < 0.001***	2137.8 ± 21.1 2443.6 ± 67.9†††	2250.1 ± 56.5 2012.1 ± 130.6†‡	H M H × M	102.233*** 0.200 28.101***	0.000(0.011) 0.654(0.000) 0.000(0.003)	1.000 0.073 1.000
Carbohydrate intake (kcal)	Non-MetS MetS *p*-value	1173.0 ± 11.2 1236.5 ± 24.4 0.021*	1170.8 ± 12.2 1244.0 ± 26.2††	1193.3 ± 26.9 1174.9 ± 85.8	H M H × M	0.237 0.252 7.335**	0.789(0.000) 0.616(0.000) 0.001(0.001)	0.087 0.079 0.939
Protein intake (kcal)	Non-MetS MetS *p*-value	324.5 ± 3.8 362.4 ± 9.8 < 0.001***	323.4 ± 4.2 367.1 ± 11.2†††	334.4 ± 9.2 323.5 ± 23.3	H M H × M	160.606*** 0.886 25.2218***	0.000(0.018) 0.347(0.000) 0.000(0.003)	1.000 0.156 1.000
Fat intake (kcal)	Non-MetS MetS *p*-value	523.9 ± 8.8 567.1 ± 23.1 0.090	517.4 ± 9.2 580.9 ± 26.6†††	581.9 ± 25.2 453.9 ± 41.2††‡	H M H × M	278.513*** 7.183** 30.312***	0.000(0.030) 0.007(0.000) 0.000(0.003)	1.000 0.764 1.000

Values are expressed as means standard errors

Main effect = H (Household) and M (Metabolic syndrome), Interaction effect = H × M (Household × Metabolic syndrome)

Two-way analysis of variance following Bonferroni post-hoc test

*MetS* metabolic syndrome

^*^p < 0.05

^**^p < 0.01

^***^p < 0.001

^†^*p* < 0.05

^††^*p* < 0.01

^†††^*p* < 0.001 compared with Non-MetS values

^‡^*p* < 0.01 compared with Multiple values

## Discussion

This study investigated differences in PA levels and energy intake by household-type and the presence of MetS in a young adult Korean population, to understand the relationships among these factors. We found that components of MetS, such as a large waist circumference, hyperlipidemia, low HDL-C levels, and high fasting blood glucose levels, can be improved by higher PA levels (Very active: 2775.1 ± 100.2 MET min/week) as compared with inactivity (0–249 MET min/week). Low HDL-C levels could also be improved by increased PA levels (Active: 500–999 MET min/week) as compared with inactivity. In addition, hyperlipidemia, low HDL-C, and high glucose levels can be improved by lower energy intake (0.8 < EER) as compared with higher energy intake (EER > 1.2). Large waist circumference and high glucose were also improved in those with lower energy intake as compared with those with moderate energy intake (0.8 ≤ EER ≤ 1.2). In the total group, there was no significant difference between MetS and non-MetS groups in terms of PA levels. This result was similar to that in multi-person households, except for the “Occupational vigorous” category. However, in single-person households, the MetS group had lower levels of “Transport” and “Total physical activity” than the non-MetS group. Investigating the differences in energy intake according to the presence or absence of MetS and according to household type showed that, in the total group, the MetS group had significantly higher total energy intake, carbohydrate intake, and protein intake levels than the non-MetS group. In multi-person households, the MetS group had significantly higher total energy intake, carbohydrate intake, fat intake, and protein intake than the non-MetS group. However, in single-person households, the MetS group had significantly lower total energy intake and fat intake than the non-MetS group. In particular, individuals with MetS who lived in single-person households had lower total energy intake and fat intake than those with MetS who lived in multi-person households. Taken together, our results showed the need for different approaches of implementing PA and nutrition strategies according to household type in order to prevent MetS.

Our results contribute to emerging evidence that PA levels and energy intake are associated with MetS components [22, 23]. We found that the prevalence of MetS factors was significantly lower among those with “Very active” PA levels as compared to those with an “Inactive” PA level. Young adults who were very active had a 45% lower prevalence of MetS. Similar to our study, Sisson et al. [24] found that men and women with higher levels of sedentary behavior than physical activity in the US had a 66% higher prevalence of MetS. Those with moderate physical activity (75–180 min/day) had a 29% lower prevalence of MetS, according to data of 4327 adults obtained in the NHANES from 2007 to 2010 [25]. Moreover, among 410 subjects, aged 18–74 years, subjects with heavy PA levels (based on the Lipid Research Clinics questionnaire) had a 40% lower prevalence of MetS than subjects with light PA levels [26]. Regular PA has been shown to increase HDL-C in a linear dose-response manner [27]. In addition, intense PA levels could reduce triglyceride levels [27]. These findings confirmed that MetS risk factors are driven largely by PA levels, and the sedentary lifestyle increases the risk of developing Mets in adults.

With respect to energy intake, we found that the MetS risk factors had significantly higher values in individuals with moderate or higher energy intake (vs. those with a lower energy intake). Those with a moderate energy intake had a 46% higher prevalence of having a large waist circumference and a 23% higher prevalence of having high fasting glucose levels. Moreover, those with a higher energy intake had a 33% higher prevalence of high triglyceride, 23% lower prevalence of low LDL-C, and 39% higher prevalence of high fasting glucose levels. Similar to our study, 7081 men aged 30 years and older, who overate more than 4 times a week, had a 141% higher prevalence of MetS [28]. According to the data from the 2007–2012 KNHANES, from 20,515 Korean adults, a high carbohydrate intake (380.8 ± 4.7 g) resulted in a 32% higher prevalence of elevated TG and a 32% higher prevalence of MetS in men, and a 26% higher prevalence of elevated TG and 31% higher prevalence of MetS in women [29]. Moreover, according to a cross-sectional study (6737 males and 8845 females) from the 2008–2011 KNHANES, a high carbohydrate intake was associated with a higher prevalence of MetS in males, and a high carbohydrate intake combined with a low fat intake was associated with MetS in females [23]. In addition, according to a study of 3-year KNHANES data, from 2012 to 2014, the prevalence of MetS was positively correlated with carbohydrate intake in an adult population, as assessed by the 24-h recall questionnaire [30]. These findings confirm that MetS is driven largely by a high nutrient energy intake, particularly that derived from carbohydrates, as young individuals with MetS had a higher carbohydrate intake than those without MetS in this study.

We found that there was no significant difference in PA levels between those with and those without MetS in the total group. However, the MetS group had a higher total energy intake, carbohydrate intake, and protein intake than those without MetS. The results of our study were very similar to those of previous studies on old adults. A higher percentage of energy intake was associated with a higher incidence of MetS, mostly due to abdominal obesity and hypertriglyceridemia in old adults [31, 32]. The prevalence of MetS has increased rapidly in Asia in recent years, and several studies have demonstrated stronger associations between dietary carbohydrate intake and metabolic disease [33, 34]. The 2007–2012 National Health and Nutrition Examination Survey studies showed that a high carbohydrate intake is associated with metabolic abnormalities (Ha et al., 2018). According to a cross-sectional study performed in 2018 (6737 males and 8845 females), the risk of MetS increased proportionally with the carbohydrate intake proportion [29]. As Korean adults consume more carbohydrates than adults in other regions, stronger associations of dietary carbohydrate with MetS were observed in this study. In addition, studies on the Korean population and a meta-analysis of observational studies have revealed that total, red, and processed meat consumption is associated with a high risk of MetS [35]. Additionally, in a review of epidemiological evidence, dietary protein appeared to increase the risk of MetS [36]. Korean eating habits have changed from a traditional diet focused on vegetables to a western diet focused on meat in recent years. Taken together, MetS in young individuals is more likely to be linked to energy intake than to PA levels.

This study analyzed household-specific PA according to the presence or absence of MetS in young adults. There was no significant difference across PA categories in multi-person households except for the “Occupational vigorous” category. In contrast, transport and total PA were significantly lower in the MetS group than in the non-MetS group in single-person households. Numerous studies in recent decades have shown that higher PA levels have a favorable impact on each of the MetS components [37–39]. In a cross-sectional evaluation of PA and metabolic risk among individuals with a family history of type 2 diabetes, it was suggested that increasing the total amount of PA in sedentary and overweight individuals had beneficial effects on MetS risk [40]. An analysis of MetS in Korean adults, identified MetS risk factors as a lack of walking and flexibility exercises in single-person households [41]. Although living alone does not equate to a lack of a social network or support, social support is important for engaging in healthy behaviors, including PA [42, 43]. Support from family [44] and friends [45] has also been shown to correlate positively with PA. Single-person households without social support could promote a decrease in total PA, which can serve as a potential risk factor for an increased incidence of MetS.

This study also analyzed household-specific dietary intake according to the presence or absence of MetS in young adults. The total energy intake, carbohydrate intake, protein intake, and fat intake were significantly higher in the MetS group than in the non-MetS group in indivuals living in multi-person households. In contrast, the total energy intake and fat intake were significantly lower in the MetS group than in the non-MetS group in individuals living in single-person households. Morever, the total energy intake and fat intake were significantly lower in individuals with MetS who lived alone than in those with MetS who lived in multi-person households. Multi-person households presented a similar tendency to the total group in terms of energy intake: those with MetS tended to have a higher energy intake, as mentioned above, whereas those living alone presented the opposite tendency. According to the 2020 Dietary Reference Intakes for Koreans [46], the recommended energy intake for men aged 19–49 is about 2550 cal, and for women aged 19–49, it is about 1950 cal. The single-person household data in this study represented both men and women. Thus, they consumed relatviely lower amounts of energy than recommended by the guidelines, but were not at a deficient level. Thus, we cautiously speculate that MetS in young adults in single-person households is more likely to be linked to lower PA levels than to energy intake.

MetS is caused by lifestyle factors, such as PA, diet, and weight, and it is reported that the risk of MetS can be reduced by increasing PA levels and by engaging in balanced eating habits [47–49]. In this study, we found that there are household-specific aspects to the PA levels and energy intake according to the presence or absence of MetS in young adults. This information can be used as a basis for preparing countermeasures through education on PA and nutrition, and by providing nutrition guidance for young adults with MetS according to the household type. Such practical measures are urgently needed to reduce the incidence of MetS among young Korean adults.

This study’s results should be interpreted with consideration of the following limitations. First, we evaluated young adults with MetS, but did not consider the timing of MetS development or the duration of MetS. Second, the amount of PA was not assessed using heart rate measurements or using an accelerometer, but was quantified based on survey findings, which are prone to errors. Third, this study reported simple differences without identifying the causality underlying the relationships between PA and nutrition. Finally, the data generated by using the 24-h-recall may not represent long-term dietary habits. Twenty-four-hour recall is essentially a retrospective method of diet assessment, where an individual is interviewed about their food and beverage consumption during the previous day or the preceding 24 h. However, a single 24-h-recall may not be representative of the habitual diet at an individual level. Accordingly, in this study, a total of 467 low-reporters and 47 over-reporters were found among females, and a total of 264 low-reporters and 62 over-reporters were found among males. Nevertheless, the strength of this study was that it analyzed the PA levels and energy intake of single-person households according to the prevalence of MetS among young adults for the first time. Most of the previous studies that investigated the relationship between metabolic syndrome, PA levels, and energy intake mainly targeted old adults. In particular, this study classified them by the types of households and investigated the PA levels and energy intake of single-person households. Therefore, our results can contribute to the ongoing research on the relationship between MetS and health behaviors.

## Conclusions

According to the ORs of MetS components, statistically significant associations were observed among low PA levels, high energy intake, and MetS components, including large waist circumference, high TG, low HDL-C, and high fasting glucose levels in young Korean adults. However, our results showed different patterns of associations, according to the household type. In multi-person households, there was no significant difference between individuals with and without MetS, although young adults with MetS had higher energy intake than without MetS. On the other hand, in single-person households, young adults with MetS had lower PA levels and lower energy intake than those without MetS. These findings highlight the need for different approaches of implementing PA and nutrition strategies according to the household type, in order to prevent MetS. Programs aimed at decreasing energy intake are necessary for multi-person households, while programs aimed at increasing PA levels and fat intake and balanced nutrition are needed for single-person households. Current trends to use personal devices, smartphones, activity trackers to measure physical activity, and applications to measure energy intake can provide innovative opportunities for researchers and healthcare providers to educate clients and intervene in new ways to promote healthy lifestyles.

## Data Availability

The dataset can be downloaded from Korea National Health and Nutrition Examination Survey website (https://www.kdca.go.kr/index.es?sid=a3).
